# Association Between Alkaline Phosphatase and Muscle Mass, Strength, or Physical Performance in Patients on Maintenance Hemodialysis

**DOI:** 10.3389/fmed.2021.657957

**Published:** 2021-05-17

**Authors:** Seok Hui Kang, Jun Young Do, Jun Chul Kim

**Affiliations:** ^1^Division of Nephrology, Department of Internal Medicine, College of Medicine, Yeungnam University, Daegu, South Korea; ^2^Division of Nephrology, Department of Internal Medicine, CHA Gumi Medical Center, CHA University, Gumi, South Korea

**Keywords:** alkaline phosphatase, hemodialysis, muscle mass, muscle strength, physical performance

## Abstract

**Introduction:** Alkaline phosphatase (ALP) is an indicator for checking liver or bone disorders, but recent studies have shown the possibility of an additive indicator beyond the simple mineral-bone status in dialysis patients. The aim of the study was to evaluate the ALP level and various indicators for malnutrition, physical performance, or hospitalization in patients on hemodialysis (HD).

**Methods:** This study was an observational study (*n* = 84). We included all patients undergoing HD with the following criteria: age ≥ 20 years, duration of dialysis ≥ 6 months, ability to ambulate without an assistive device, ability to communicate with the interviewer, and no hospitalization within the last 3 months before enrollment. Furthermore, none of the patients had liver disease. We recommended abstinence of alcohol for ≥ 1 month for the duration of the study. The patients were divided into tertiles based on the ALP level. Muscle mass [appendicular muscle mass index using dual-energy X-ray absorptiometry (ASM/Ht^2^), thigh muscle area index using computed tomography (TMA/Ht^2^)], strength [handgrip strength (HGS)], and physical performance [gait speed (GS), sit-to-stand for 30-s test (STS30), 6-min walk test (6-MWT), or Short Physical Performance Battery test (SPPB)] were evaluated. The number of hospitalizations was also evaluated.

**Results:** The ALP level in the low, middle, and high tertiles was 50.5 ± 7.5, 69.8 ± 5.4, and 113.3 ± 47.3 IU/l, respectively. The high tertile group showed the poorest trends in ASM/Ht^2^, TMA/Ht^2^, HGS, GS, STS30, and 6-MWT compared to the other tertile groups. Logistic regression analysis showed that the high tertile group for low HGS, low GS, or low SPPB had a higher odds ratio compared to the other tertiles. Subgroup analyses according to age, sex and diabetes mellitus showed similar trends as in the total cohort. Hospitalization-free survival rates after 300 days in the high tertile and the other tertiles were 53.8 and 77.2%, respectively (*P* = 0.105).

**Conclusion:** The present study demonstrated that ALP is associated with muscle mass, strength, and physical performance in patients on maintenance HD. In addition, the trend showed better hospitalization-free survival in the low or middle tertiles than in the high tertile. ALP can be considered as a simple and useful indicator to detect malnutrition, physical performance, or hospitalization in patients on HD.

## Introduction

End-stage renal disease is the most advanced stage of chronic kidney disease and requires renal replacement therapy, such as hemodialysis (HD), peritoneal dialysis, or kidney transplantation. HD is an important dialysis modality performed in more than 80% of patients with end-stage renal disease (1, 2). Short-term mortality continues to decrease due to the improvements in dialysis technology; however, long-term complications continue to increase. Malnutrition combined with a decrease in muscle mass or physical performance is an emerging concern in patients on long-term HD (3). These are associated with a decrease in the quality of life and an increase in morbidity or mortality. Some of conventional methods for predicting malnutrition in HD patients are serum albumin, body mass index (BMI), dual-energy X-ray absorptiometry (DXA), or various laboratory indicators (4). However, none of them can be considered as a gold-standard for predicting malnutrition because patients on HD have specific conditions such as hypervolemia or chronic kidney disease-mineral bone disease (CKD-MBD) (4, 5). Attempts at discovering easier and/or more economical methods for evaluating malnutrition in clinical practice continue.

Serum alkaline phosphatase (ALP) is originally a hydrolytic enzyme that removes phosphates from various molecules such as protein and nucleotides (6). It is an indicator for checking liver or bone disorders and is commonly evaluated in clinical practice. For patients on HD, Kidney Disease: Improving Global Outcomes recommends monitoring serum ALP activity to evaluate CKD-MBD (7). However, recent studies have shown the possibility of an additive indicator beyond the simple CKD-BMD status. Kim et al. showed the association between ALP level and vascular calcification (8). A previous study among non-dialysis patients showed that ALP is associated with body fat mass and atherogenic lipid profile (9). Other studies showed an association between ALP and inflammatory indicators (10, 11). However, there are few studies about the association between ALP level and nutritional indicators with comprehensive data, including muscle mass indices, strength, and laboratory and physical performance measurements in patients on HD. The aim of the study was to evaluate the ALP level and various indicators, physical performance, or hospitalization for malnutrition.

## Subjects and Methods

### Study Population

This study was performed in a tertiary medical center between September 2012 and March 2015. It was an observational study based on the analysis of an existing data-set (12). We included all patients undergoing HD aged ≥ 20 years, duration of dialysis ≥ 6 months, ability to ambulate without an assistive device, ability to communicate with the interviewer, and no hospitalization within the last 3 months before enrollment. None of the patients were on opioids, antihistamines, or antidepressants, which are associated with decreased physical activity and cognitive function. Furthermore, none of the patients had acute/chronic viral hepatitis, fatty, or alcoholic liver disease. We recommended abstinence of alcohol for ≥1 month before laboratory, radiological, or physical performance measurements. A total of 84 patients were enrolled. The patients were divided into tertiles based on the ALP level as low, middle, and high. This study was approved by the institutional review board of a Medical Center (approval no. 12-07). The board waived the need for informed consent because the subjects' records and information were anonymized and de-identified prior to the analysis.

### Baseline Variables

Collected baseline data were sex, age, presence of diabetes mellitus (DM), dialysis vintage, hemoglobin (g/dl), C-reactive protein (CRP, mg/dl), blood urea nitrogen (mg/dl), creatinine (mg/dl), aspartate transaminase (U/l), alanine transaminase (U/l), calcium (mg/dl), phosphorus (mg/dl), sodium (mEq/l), potassium (mEq/l), chloride (mEq/l), intact-parathyroid hormone (i-PTH, pg/ml), 25-hydroxy (25-OH) vitamin D (ng/ml), total cholesterol (mg/dl), albumin (g/dl), and single-pool Kt/Vurea (spKt/Vurea). All laboratory findings were performed prior to the HD sessions and were repeated thrice in the following 3 weeks. The mean of three values was considered for each parameter. DM was defined as a patient-reported history and a medical record of a DM diagnosis or medication. spKt/Vurea was calculated using the described in a previous study (13).

### Assessment of ALP, Muscle Mass, Strength, and Subjective Global Assessment Score

ALP level (IU/l) was measured in the midweek before the HD session, and the measurements were repeated thrice during 3 weeks. The mean ALP of the three values was considered. ALP was measured using Bayer Reagent Packs on an automated chemistry analyzer (Advia 1650 Autoanalyzer; Bayer Diagnostics, Leverkusen, Germany; ALP normal adult range 45-129 IU/l).

BMI (kg/m^2^) was calculated as body weight divided by the square of height in meters. Hand grip strength (HGS) was measured in all patients. Each patient performed three trials with the dominant hand using a manual hydraulic dynamometer (Jamar® Hydraulic hand dynamometer; Sammons Preston, Chicago, IL, USA). Maximum values among the three trials were selected. Subjective global assessment (SGA) was calculated based on scores from seven items (weight loss, dietary intake, gastrointestinal symptoms, functional capacity, comorbidity, decreased fat, and decreased muscle mass) (14). The thigh muscle area (TMA, cm^2^) was calculated using the mid-thigh computed tomography scan using a 320-slice CT scanner (Aquilion ONE; Toshiba Medical Systems Corp., Tokyo, Japan). An axial image was obtained at the midpoint of a line extending from the superior border of the patella to the greater trochanter (3 mm thickness, five slices). The images were analyzed using an image analysis software (ImageJ 1.45S; National Institutes of Health, Bethesda, MD, USA). Finally, TMA/Ht^2^ was calculated as TMA divided by the square of height in meters.

In addition, total fat mass (FM) and total bone mineral density (BMD, g/cm^2^) were evaluated using whole-body DXA (GE Medical Systems Lunar, Madison, WI, USA). Visceral fat area (VFA) was obtained from multi-frequency bioimpedance analysis (Inbody, Seoul, Korea). These measurements were performed in the midweek following the HD session. Total FM index from DXA was calculated as total FM divided by the square of height in meters. In addition, using reactance (Xc) and resistance (R) obtained from bioimpedance analysis at 50 kHz, phase angle (PhA) was estimated using the formula: PhA (°) = arctangent (Xc/R) × (180/p).

### Assessment of Physical Performance and Hospitalization

Gait speed (GS, m/s) was evaluated using the timed 4-m walking test (15). For the 5 times sit-to-stand test (5STS), each patient was seated on a chair with the arms crossed and the hands touching the shoulders (16). The patients were asked to stand up and sit down 5 times as quickly as possible, and the time taken in seconds was recorded. For the 30-s sit-to-stand test (STS30), the patients were seated on a chair with the arms crossed and the hands touching the shoulders. Scores were defined as the number of stand-ups a patient could complete in 30 s without using the arms as support (17). For the 6-min walk test (6-MWT, m), the patients were asked to walk at their usual pace for 6 min, and the distance covered was recorded in meters (18). For the timed up and go test (TUG, s), the patients were instructed to stand up from an arm-chair, walk 3 m, turn around, return to the chair, and sit down (19). The time in seconds was recorded. Short Physical Performance Battery test (SPPB) was calculated using GS, 5STS, 6-MWT, and balance test, with a score between 0 and 12 (20). In addition, a low group was defined based on their values on the SPPB, HGS, and GS tests, according to previous studies (15, 21). The low SPPB group was defined as participants with a score of ≤10 (21). The low GS group was defined as those with a score of ≤0.8 m/s, and the low HGS group was defined as participants <26 kg in men and <18 kg in women. Finally, the number of hospitalizations, regardless of the causes, was evaluated at the end-point of follow-up (follow-up interval: 63–939 days). HGS, SGA, TMA, DXA measurements, and physical performance tests were all evaluated following the midweek HD day.

### Statistical Analysis

The data were analyzed using the statistical software IBM SPSS Statistics version 25 (SPSS Inc., Chicago, IL, USA). Categorical variables were expressed as counts (percentages). Continuous variables were expressed as mean ± standard deviation. For continuous variables, means were compared using the one-way analysis of variance, followed by *post-hoc* Tukey comparison. The correlation between two continuous variables was assessed using Pearson's or partial correlation analyses. Linear regression analysis was performed to assess the independent predictors of ASM/Ht^2^, TMA/Ht^2^, SGA, serum albumin, 5STS, STS30, 6-MWT, TUG, or total BMD. Logistic regression analysis was performed to assess the independent predictors of low HGS, low GS, or low SPPB groups. Multivariate analysis was performed for variables with *P* < 0.100 on univariate analyses among age, sex, presence of DM, i-PTH, 25-(OH) vitamin D, or ALP levels. Kaplan-Meier analysis was used to plot survival among the groups, and the Beslow method was used to obtain statistical significance. The level of statistical significance was set at *P* < 0.05.

## Results

### Participants' Clinical Characteristics

The ALP level in the low, middle, and high tertiles was 50.5 ± 7.5 (interval, 35–59), 69.8 ± 5.4 (interval, 61–78), and 113.3 ± 47.3 (interval, 79–277) IU/l, respectively. The mean age in the low, middle, and high tertiles was 55.8 ± 12.8, 56.8 ± 12.5, and 56.9 ± 10.9 years, respectively (Table 1). The i-PTH level was significantly lower in the low tertile group than in the other tertiles. There were no significant differences in dialysis vintage, presence of DM, and laboratory findings, except i-PTH, among the three tertiles. The numbers of patients taking vitamin D agents were 12 (42.9%) in the low tertile, 17 (60.7%) in the middle tertile, and 22 (78.6%) in the high tertile groups (*P* = 0.021). In our cohort, SGA score and serum albumin were 5.7 ± 1.0 and 3.8 ± 0.3 g/dl, respectively. BMI, ASM/Ht^2^, TMA/Ht^2^, and total BMD were 23.7 ± 3.6 kg/m^2^, 6.6 ± 1.0 kg/m^2^, 36.9 ± 7.0 cm^2^/m^2^, and 1.06 ± 0.13 g/cm^2^, respectively. HGS, GS, SPPB, 5STS, STS30, 6-MWT, and TUG were 26.0 ± 7.4 kg, 0.9 ± 0.2 m/s, 10.9 ± 1.6, 8.9 ± 6.2 s, 17.8 ± 5.7, 459 ± 113 m, and 7.3 ± 2.0, respectively.

**Table 1:** Demographic and laboratory findings according to tertiles of alkaline phosphatase. Participants' clinical characteristics.

	**Low tertile (*n* = 28)**	**Middle tertile (*n* = 28)**	**High tertile (*n* = 28)**	***P*-value**
Sex (male, %)	10 (35.7%)	13 (46.4%)	17 (60.7%)	0.171
Age (years)	55.8 ± 12.8	56.8 ± 12.5	56.9 ± 10.9	0.926
Diabetes mellitus (%)	14 (50%)	12 (42.9%)	18 (64.3%)	0.263
Dialysis vintage (years)	3.9 ± 4.8	4.5 ± 4.4	5.2 ± 6.2	0.639
Hemoglobin (mg/dL)	11.1 ± 0.6	10.9 ± 0.6	10.9 ± 0.6	0.230
Blood urea nitrogen (mg/dl)	57.9 ± 12.2	57.2 ± 13.0	63.2 ± 17.9	0.246
Creatinine (mg/dl)	10.2 ± 2.5	10.6 ±.7	10.0 ± 2.7	0.667
Aspartate transaminase (U/l)	18 ± 5	17 ± 5	19 ± 7	0.664
Alanine transaminase (U/l)	15 ± 5	16 ± 8	16 ± 9	0.938
Serum calcium (mg/dl)	8.5 ± 0.7	8.3 ± 0.8	8.4 ± 0.6	0.649
Serum phosphorus (mg/dl)	5.3 ± 1.2	5.6 ± 1.3	5.3 ± 1.3	0.691
Serum sodium (mEq/l)	137 ± 3	138 ± 2	137 ± 3	0.398
Serum potassium (mEq/l)	4.9 ± 0.7	5.0 ± 0.5	5.1 ± 0.5	0.291
Serum chloride (mEq/l)	98 ± 3	99 ± 3	98 ± 4	0.360
Intact parathyroid hormone (pg/ml)	145 ± 113	283 ± 139^*^	362 ± 215^*^	<0.001
Total cholesterol (mg/dl)	156 ± 30	143 ± 28	162 ± 42	0.107
25-hydroxy vitamin D	12.3 ± 8.5	12.4 ± 8.0	9.7 ± 4.0	0.278
spKt/Vurea	1.3 ± 0.2	0.4 ± 0.4	1.4 ± 0.2	0.442

*Data are expressed as mean ± standard deviation for continuous variables and as number (percentage) for categorical variables. P-values were tested using one-way analysis of variance, followed by a post-hoc Tukey comparison for continuous variables and Pearson's χ^2^ or Fisher's exact tests for categorical variables*.

* *P < 0.05 compared with low tertile*.

### Association Between ALP Tertiles and Body Composition, Strength, SGA Score, Physical Performances, or Hospitalization

**Figure 1:**
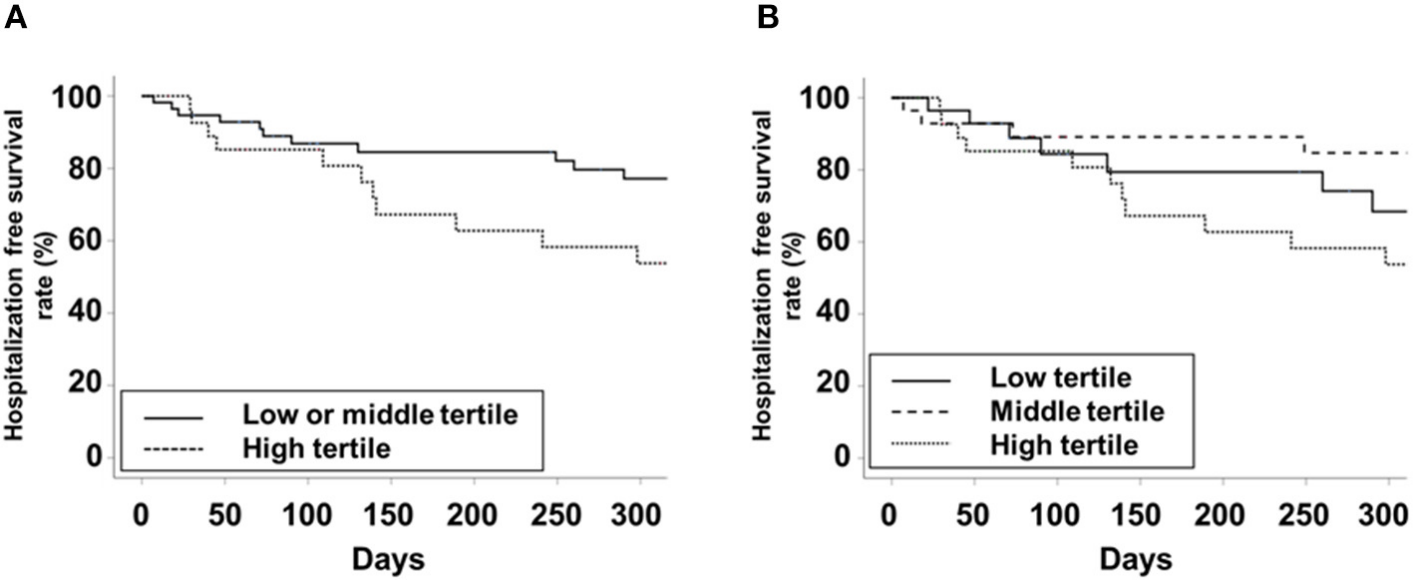
Kaplan-Meier curve for hospitalization-free survival. **(A)** Low or middle vs. high tertiles. **(B)** low vs. middle vs. high tertiles.

The high tertile group showed the poorest trends in ASM/Ht^2^, TMA/Ht^2^, BMI, total BMD, HGS, GS, STS30, and 6-MWT compared to the other tertile groups on univariate analysis (Table 2). ALP level as a continuous variable had a significant correlation with ASM/Ht^2^, TMA/Ht^2^, BMI, SGA score, total BMD, HGS, GS, STS30, 6-MWT, and TUG (Supplementary Table 1). The i-PTH and 25-(OH) vitamin D levels did not show a more superior association with muscle mass, strength, and physical performance compared to ALP. Linear regression analysis showed that ALP was inversely associated with ASM/Ht^2^, TMA/Ht^2^, SGA score, or STS30 on univariate and multivariate analyses (Supplementary Table 2). 6-MWT, TUG, and total BMD had a significant association with ALP on univariate analysis alone. There were no significant associations between 5STS or serum albumin and ALP levels. Logistic regression analysis showed that the high tertile group for low HGS, low GS, or low SPPB had a higher odds ratio compared to the other tertiles (Table 3). In addition, we performed correlation analysis between PhA and ALP levels, and a significant correlation between them was obtained (*r* = −0.339, *P* = 0.002). The ALP levels in patients with < 5° or ≥ 5° for phase angle were 83.5 ± 42.8 and 67.8 ± 24.5 IU/L, respectively (*P* = 0.037). Hospitalization-free survival rates in the high tertile and the other tertiles were 53.8 and 77.2% in 300 days (Figure 1A, *P* = 0.105). Those in the low tertile and the middle tertiles were 68.4 and 84.7%, respectively, in 300 days (Figure 1B, *P* = 0.289). The total number of hospital admissions in the high or other tertiles were 1.36 ± 2.04 and 0.71 ± 1.42, respectively (*P* = 0.097). Although there was no statistical significance, the trend showed better hospitalization-free survival in low or middle tertiles than in the high tertile.

**Table 2:** Comparison of muscle mass indices, nutritional and physical performance according to tertiles of alkaline phosphatase.

	**Low tertile**	**Middle tertile**	**High tertile**	***P*-value**
ASM/ Ht^2^ (kg/m^2^)	6.7 ± 1.1	6.9 ± 0.8	6.2 ± 0.8^#^	0.020
TMA/Ht^2^ (cm^2^/m^2^)	38.0 ± 8.1	38.9 ± 4.9	33.8 ± 6.9^#^	0.013
Body mass index (kg/m^2^)	24.6 ± 3.9	24.3 ± 3.4	22.3 ± 3.3^*^	0.034
SGA score	5.9 ± 1.0	5.8 ± 1.1	5.4 ± 1.0	0.161
Serum albumin (mg/dL)	3.8 ± 0.3	3.8 ± 0.3	3.9 ± 0.3	0.742
Total BMD (g/cm^2^)	1.13 ± 0.13	1.07 ± 0.10	0.99 ± 0.10^*^^#^	<0.001
Handgrip strength (kg)	25.6 ± 6.6	29.6 ± 7.1	22.8 ± 7.0^#^	0.002
Gait speed (m/s)	0.95 ± 0.22	0.97 ± 0.19	0.83 ± 0.15^#^	0.018
SPPB	10.7 ± 2.1	11.3 ± 1.2	10.6 ± 1.4	0.254
5STS (s)	10.2 ± 10.0	7.1 ± 2.0	9.3 ± 2.5	0.149
STS30	17.0 ± 5.8	20.8 ± 5.4^*^	15.7 ± 4.9^#^	0.002
6-MWT (m)	464 ± 131	496 ± 106	418 ± 87^#^	0.032
Timed up-to-go test	7.4 ± 2.4	6.6 ± 1.6	7.9 ± 1.8	0.057

*Data are expressed as mean ± standard deviation. P-values were tested using one-way analysis of variance, followed by a post-hoc Tukey comparison*.

* *P < 0.05 compared with low tertile and*

# *P < 0.05 compared with middle tertile. ASM/Ht^2^, appendicular skeletal muscle mass per height squared; TMA/Ht^2^, thigh muscle area per height squared; SGA, subjective global assessment; BMD, bone mineral density; SPPB, short physical performance battery; 5STS, 5 times sit-to-stand test; STS30, the 30-s sit-to-stand test; 6-MWT, 6-min walk test*.

**Table 3:** Logistic regression analysis of low HGS, GS, or SPPB according to variables.

	**Univariate**		**Multivariate**	
	**OR (95% CI)**	***P*-value**	**OR (95% CI)**	***P*-value**
**Dependent variable: low HGS**				
Age	1.04 (0.99–1.09)	0.178	–	–
Sex (ref: men)	0.96 (0.31–2.92)	0.935	–	–
Diabetes mellitus	2.06 (0.64–6.65)	0.277	–	–
Intact parathyroid hormone	1.00 (1.00–1.00)	0.899	–	–
25-(OH) vitamin D	0.87 (0.75–1.01)	0.069	0.99 (0.93–1.06)	0.793
ALP (ref: low or middle tertile)	3.95 (1.24–12.59)	0.020	4.73 (1.43–15.62)	0.011
**Dependent variable: low GS**				
Age	1.05 (1.01–1.09)	0.024	1.05 (1.01–1.10)	0.025
Sex (ref: men)	4.71 (1.72–12.91)	0.003	4.51 (1.51–13.45)	0.007
Diabetes mellitus	1.29 (0.52–3.17)	0.585	–	–
Intact parathyroid hormone	1.00 (1.00–1.01)	0.168	–	–
25-(OH) vitamin D	0.98 (0.92–1.04)	0.460	–	–
ALP (ref: low or middle tertile)	4.84 (1.48–15.78)	0.009	4.40 (1.26–15.45)	0.021
**Dependent variable: low SPPB**				
Age	1.04 (1.00–1.08)	0.075	1.04 (0.99–1.09)	0.091
Sex (ref: men)	1.02 (0.40–2.61)	0.964	–	–
Diabetes mellitus	4.31 (1.50–12.35)	0.007	3.81 (1.27–11.42)	0.017
Intact parathyroid hormone	1.00 (1.00–1.00)	0.532	–	–
25-(OH) vitamin D	0.97 (0.90–1.05)	0.440	–	–
ALP (ref: low or middle tertile)	3.18 (1.19–8.46)	0.021	2.93 (1.03–8.37)	0.045

*Multivariate analysis was adjusted for variables with P < 0.100 on univariate analysis*.

*OR, odds ratio; CI, confidence interval; HGS, handgrip strength; GS, gait speed; SPPB, short physical performance battery; 25-(OH) vitamin D, 25-hydroxy vitamin D; ALP, alkaline phosphatase*.

### Subgroup Analyses According to Age, Sex, and DM

We divided the subjects into two age groups using an age cutoff of 65 years. In those aged < 65 years, most variables, except BMI, SGA score, serum albumin, and SPPB, showed significant association with the ALP level (Supplementary Table 3). Statistical significance for the variables was weak in those aged ≥ 65 years; however, the trends were similar to those in the age < 65 years group. On analyses by sex or the presence of DM, the overall associations were higher in women or patients without DM than in men or patients with DM.

### Association Between ALP and CRP or Fat Mass

The VFA in low, middle, and high tertiles was 104.9 ± 30.4, 99.8 ± 24.2, and 85.3 ± 24.1 cm^2^, respectively (*P* = 0.019). Total FM index (kg/m^2^) in low, middle, and high tertiles was 7.3 ± 3.3, 6.9 ± 3.0, and 5.9 ± 2.7 kg/m^2^, respectively (*P* = 0.059). The CRP levels in the low, middle, and high tertiles were 0.26 ± 0.23, 0.28 ± 0.31, and 0.73 ± 0.90, respectively (*P* = 0.003). Correlation coefficients with ALP were 0.184 for CRP (*P* = 0.094), −0.236 for VFA (*P* = 0.031), and −0.089 for total FM index (*P* = 0.423), respectively. Partial correlation coefficients after adjusting for age, sex, and DM were 0.227 for CRP (*P* = 0.041), −0.234 for VFA (*P* = 0.035), and −0.179 for total FM index (*P* = 0.109), respectively.

## Discussion

In our study, a high tertile of ALP was associated with muscle mass indices, strength, and physical performances on univariate and multivariate analyses. The high tertile of ALP was associated with higher i-PTH level and intake of vitamin D agents; however, the ALP showed the highest correlation with muscle mass indices, strength, and physical performance compared to i-PTH or 25-(OH) vitamin D levels. Further, the hospitalization-free survival rate was poorer in the high tertile than in the other tertiles; however, there was no statistical significance. Subgroup analyses showed similar trends as in the total cohort; however, the statistical significance was higher in those aged < 65 years, women, and non-DM subjects. In addition, ALP was inversely associated with VFA and positively associated with CRP level.

Two previous studies with large sample sizes showed poor survival in patients with high ALP levels on maintenance HD (6, 10). In patients on HD, high ALP is associated with CKD-MBD, such as high i-PTH level. Therefore, previous studies have focused on the correlation between high ALP level and cardiovascular disease, based on the association between ALP and vascular calcification, smooth muscle remodeling, or as a bystander of CKD-MBD. However, Zhu et al. performed a longitudinal study and did not show a significant association between ALP level and survival (22). Beddhu et al. showed the association of ALP level with all-cause mortality but not with cardiovascular events (23). A meta-analysis of 12 studies concluded that the all-cause mortality was higher in patients with high ALP levels than in those with low ALP; however, there was a negative association between high ALP and cardiovascular mortality in HD patients (24). These results reveal that other complications beyond cardiovascular events might be associated with high mortality in patients with high ALP levels.

ALP can be positively correlated with metabolic or nutritional disturbances. Kim et al. investigated the association between ALP level and increased fat in the general population (9). Beberashvili et al. performed a longitudinal study and showed the positive association between ALP level and nutritional or inflammatory indicators beyond simple CKD-MBD markers (25). These two studies hypothesized the relationship between ALP and inflammation. Other studies also showed a positive association between ALP and CRP levels in patients on HD (10, 11). Our results reveal an identical trend in the association between ALP and CRP levels, but an inverse association between ALP and fat mass. VFA decreased as the tertile of ALP increased in our study. The total FM index also showed a similar trend. These results reveal that high ALP level in patients on HD is more likely to be associated with malnutrition and is different from the association between high ALP level and insulin resistance combined with high fat mass in the general population. In the general population, a physiologic decrease in muscle mass is a known aging process and leads to a decrease in muscle mass combined with increased fat mass. However, in patients on HD, the decrease in muscle is a pathologic process caused by chronic inflammation and leads to a decrease in both muscle mass and fat mass. Our results are in line with these pathologic changes.

Although our findings are consistent with some results from previous studies that have demonstrated the association between ALP level and nutritional markers, few studies also included accurate and comprehensive measurements, such as laboratory findings, muscle mass measurements using DXA or CT, muscle strength, physical performance tests, and hospitalization-free survival. Our study evaluated the overall association between ALP level and body composition, strength, SGA score, physical performances, or hospitalization. The ALP level was significantly associated with muscle mass indices, strength, and physical performance measurements. Our data revealed that ALP level was also inversely associated with PhA. PhA is a well-known nutritional indicator associated with a healthy cell membrane, and it can be obtained using bioimpedance analysis (26). In addition, the hospitalization-free survival rate was poorer in the high tertile than in the other tertiles; however, the statistical significance was weak.

We found inverse associations between ALP level and muscle mass, strength, or physical performance, but the association with serum albumin was weak. These discrepancies might be associated with an inherent drawback of albumin as a nutritional index. Serum albumin level could be low despite a normal nutritional status due to dilution caused by hypervolemia and conditions with decreased albumin synthesis such as liver disease. In addition, mild catabolic status could be associated with normal serum albumin through metabolic adaptation in the hepatic synthesis of albumin (27). Our results reveal that patients on HD exposed to chronic inflammation might have normal serum albumin due to hepatic compensation, and clinicians might misinterpret this as normal nutritional status. Additional investigations for protein metabolism should be considered in patients on HD with normal serum albumin level to overcome these challenges.

In our study, most analyses were performed using tertile of ALP levels. Categorization for a continuous variable is helpful to evaluate its impact at different levels; however, a dichromatic approach using a low or high level for one cut-off value may have limitations, considering error of measurements or variables with a non-linear association with outcomes. Therefore, categorization using three or more groups, such as tertile or quartile, is more useful in identifying trends based on variable level. In our study, we divided the participants into tertiles due to the small sample size. In addition, we performed correlation and linear regression analyses to overcome inherent limitations in categorical group analysis, and the resulting continuous variable data were found to be similar with those using categorical groups.

Our data showed a positive association between ALP and i-PTH levels. PTH activates osteoblast and increases ALP level (28). Previous studies showed the positive association between the two variables in HD patients, but the strength of association differs according to i-PTH level (29, 30). In addition, there were different results in the association between each variable and outcomes in HD patients. Previous studies showed that there were inverse linear associations between ALP and mortality, but there were the other studies which showed non-linear or non-significant association between i-PTH and mortality (6, 31). These discrepancies, the stronger association between ALP and the outcome, were explained as follows: ALP originating from the bone itself can more likely reflect internal bone activity, there is low variability of ALP compared to i-PTH, and that there is a stronger effect of vascular calcification in ALP than i-PTH (6, 31–34).

Our study has inherent limitations, including a single center and a small number of patients. Further, this study was a retrospective analysis using a cohort from a previous study (12). We believe that the lack of statistical significance in some physical performance tests or hospitalization-free survival rate might be associated with the small number of patients or events. Second, we did not perform multivariate analyses with adjustment for all confounding factors such as calcium, phosphorus, i-PTH, CRP, liver enzymes, or 25-(OH) vitamin D levels. Multivariate analyses in our study were adjusted for some selected variables alone due to the small sample size. Third, our study did not evaluate the origin of serum ALP such as bone-specific ALP or liver-specific ALP. A prospective longitudinal study including a large number of patients and more precise ALP measurements is warranted to overcome these limitations.

In conclusion, the present study demonstrated that ALP is associated with muscle mass, strength, and physical performance in patients on maintenance HD. In addition, the trend showed better hospitalization-free survival in low or middle tertiles than in the high tertile. ALP can be considered as a simple and useful indicator to detect malnutrition, physical performance, or hospitalization in patients on HD.

## Data Availability Statement

The original contributions presented in the study are included in the article/Supplementary Material, further inquiries can be directed to the corresponding author/s.

## Ethics Statement

The studies involving human participants were reviewed and approved by the institutional review board of Gumi CHA Medical Center. The ethics committee waived the requirement of written informed consent for participation.

## Author Contributions

SK conceptualized and designed the study, performed the analysis and interpretation of data, and wrote the manuscript. JD and JK generated, collected the data, and drafted and revised the manuscript. All authors approved the final version of the manuscript.

## Conflict of Interest

The authors declare that the research was conducted in the absence of any commercial or financial relationships that could be construed as a potential conflict of interest.
