# Supplementary material for: Association Between Alkaline Phosphatase and Muscle Mass, Strength, or Physical Performance in Patients on Maintenance Hemodialysis
Source: Front Med (Lausanne). 2021 May 17;8:657957. doi: 10.3389/fmed.2021.657957 (PMC8165237; doi:10.3389/fmed.2021.657957)
Supplement: Supplementary file 1 [file Table_1.docx]

**Table S1. Correlation between alkaline phosphatase, intact parathyroid hormone, or 25(OH)-vitamin D levels and various indices**

|  | **Independent variables** | | | | | |
| --- | --- | --- | --- | --- | --- | --- |
|  | **Alkaline phosphatase** | | **Intact parathyroid hormone** | | **25-(OH) vitamin D** | |
|  | ***r*** | ***P*-value** | ***r*** | ***P*-value** | ***r*** | ***P*-value** |
| ASM/ Ht^2^ (kg/m^2^) | –0.335 | 0.002 | –0.038 | 0.733 | 0.052 | 0.642 |
| TMA/Ht^2^ (cm^2^/m^2^) | –0.331 | 0.002 | –0.078 | 0.480 | 0.081 | 0.466 |
| Body mass index (kg/m^2^) | –0.237 | 0.030 | –0.088 | 0.423 | –0.175 | 0.112 |
| SGA score | –0.285 | 0.009 | 0.095 | 0.388 | 0.036 | 0.744 |
| Serum albumin (mg/dl) | 0.030 | 0.787 | –0.003 | 0.976 | 0.201 | 0.066 |
| Total BMD (g/cm^2^) | –0.361 | 0.001 | –0.358 | 0.001 | –0.033 | 0.766 |
| Handgrip strength (kg) | –0.290 | 0.007 | –0.046 | 0.677 | 0.257 | 0.018 |
| Gait speed (m/s) | –0.309 | 0.004 | –0.118 | 0.284 | 0.158 | 0.152 |
| SPPB | –0.195 | 0.075 | 0.070 | 0.525 | 0.116 | 0.293 |
| 5STS (s) | 0.096 | 0.385 | –0.088 | 0.424 | –0.166 | 0.130 |
| STS30 | –0.299 | 0.006 | –0.048 | 0.668 | 0.223 | 0.041 |
| 6-MWT (m) | –0.246 | 0.024 | –0.005 | 0.964 | 0.205 | 0.061 |
| Timed up-to-go test | 0.242 | 0.027 | 0.046 | 0.675 | –0.201 | 0.066 |

Correlation analyses were analyzed using Pearson’s correlation.

Abbreviations: 25-(OH) vitamin D, 25-hydroxy vitamin D; ASM/Ht^2^, appendicular skeletal muscle mass per height squared; TMA/Ht^2^, thigh muscle area per height squared; SGA, subjective global assessment; BMD, bone mineral density; SPPB, short physical performance battery; 5STS, five times sit-to-stand test; STS30, 30-second sit-to-stand test; 6-MWT, 6-minute walk test.
