# Supplementary material for: Association Between Alkaline Phosphatase and Muscle Mass, Strength, or Physical Performance in Patients on Maintenance Hemodialysis
Source: Front Med (Lausanne). 2021 May 17;8:657957. doi: 10.3389/fmed.2021.657957 (PMC8165237; doi:10.3389/fmed.2021.657957)
Supplement: Supplementary file 2 [file Table_2.docx]

**Table S2. Linear regression analysis of indices according to alkaline phosphatase levels**

|  | Univariate | | Multivariate | |
| --- | --- | --- | --- | --- |
|  | Standardized β (SE) | *P*-value | Standardized β (SE) | *P*-value |
| Dependent variable: ASM/Ht^2^ |  |  |  |  |
| Age | –0.23 (0.10) | 0.033 | –0.17 (0.01) | 0.049 |
| Sex (ref: men) | –0.58 (0.17) | <0.001 | –0.53 (0.17) | <0.001 |
| DM | 0.12 (0.21) | 0.285 | – | – |
| i-PTH | –0.04 (0.00) | 0.733 | – | – |
| 25-(OH) vitamin D | 0.05 (0.02) | 0.642 | – | – |
| Alkaline phosphatase | –0.34 (0.00) | 0.002 | –0.19 (0.00) | 0.031 |
| Dependent variable: TMA/Ht^2^ |  |  |  |  |
| Age | –0.23 (0.06) | 0.034 | –0.18 (0.06) | 0.077 |
| Sex (ref: men) | –0.35 (1.45) | 0.001 | 0.28 (1.42) | 0.006 |
| DM | –0.01 (1.55) | 0.952 | – | – |
| i-PTH | –0.08 (0.00) | 0.480 | – | – |
| 25-(OH) vitamin D | 0.08 (0.11) | 0.466 | – | – |
| Alkaline phosphatase | –0.33 (0.02) | 0.002 | –0.24 (0.02) | 0.020 |
| Dependent variable: SGA score |  |  |  |  |
| Age | –0.36 (0.01) | 0.001 | –0.33 (0.01) | 0.002 |
| Sex (ref: men) | –0.09 (0.23) | 0.446 | – | – |
| DM | –0.13 (0.22) | 0.256 | – | – |
| i-PTH | 0.10 (0.00) | 0.388 | – | – |
| 25-(OH) vitamin D | 0.04 (0.02) | 0.744 | – | – |
| Alkaline phosphatase | –0.29 (0.00) | 0.009 | –0.24 (0.00) | 0.023 |
| Dependent variable: serum albumin |  |  |  |  |
| Age | –0.33 (0.00) | 0.002 | –0.32 (0.00) | 0.002 |
| Sex (ref: men) | 0.04 (0.06) | 0.712 | – | – |
| DM | 0.13 (0.06) | 0.238 | – | – |
| i-PTH | –0.00 (0.00) | 0.976 | – | – |
| 25-(OH) vitamin D | 0.20 (0.00) | 0.066 | 0.19 (0.00) | 0.072 |
| Alkaline phosphatase | 0.03 (0.00) | 0.787 | – | – |
| Dependent variable: 5STS |  |  |  |  |
| Age | 0.27 (0.06) | 0.014 | – | – |
| Sex (ref: men) | –0.05 (1.35) | 0.630 | – | – |
| DM | 0.13 (1.34) | 0.239 | – | – |
| i-PTH | –0.09 (0.00) | 0.424 | – | – |
| 25-(OH) vitamin D | –0.17 (0.09) | 0.130 | – | – |
| Alkaline phosphatase | 0.10 (0.02) | 0.385 | – | – |
| Dependent variable: STS30 |  |  |  |  |
| Age | –0.37 (0.05) | 0.001 | –0.33 (0.05) | 0.002 |
| Sex (ref: men) | –0.11 (1.25) | 0.326 | – | – |
| DM | –0.14 (1.25) | 0.222 | – | – |
| i-PTH | –0.05 (0.00) | 0.668 | – | – |
| 25-(OH) vitamin D | 0.22 (0.09) | 0.041 | 0.18 (0.08) | 0.083 |
| Alkaline phosphatase | –0.30 (0.02) | 0.006 | –0.22 (0.02) | 0.029 |
| Dependent variable: 6-MWT |  |  |  |  |
| Age | –0.47 (0.92) | <0.001 | –0.43 (0.91) | <0.001 |
| Sex (ref: men) | –0.13 (24.63) | 0.230 | – | – |
| DM | –0.21 (24.30) | 0.057 | –0.15 (21.64) | 0.117 |
| i-PTH | –0.01 (0.07) | 0.964 | – | – |
| 25-(OH) vitamin D | 0.21 (1.70) | 0.061 | 0.14 (1.52) | 0.162 |
| Alkaline phosphatase | –0.25 (0.32) | 0.024 | –0.17 (0.29) | 0.080 |
| Dependent variable: TUG |  |  |  |  |
| Age | 0.47 (0.02) | <0.001 | 0.42 (0.02) | <0.001 |
| Sex (ref: men) | 0.06 (0.45) | 0.577 | – | – |
| DM | 0.22 (0.44) | 0.043 | 0.17 (0.39) | 0.088 |
| i-PTH | 0.05 (0.00) | 0.675 | – | – |
| 25-(OH) vitamin D | –0.20 (0.03) | 0.066 | –0.13 (0.03) | 0.180 |
| Alkaline phosphatase | 0.24 (0.01) | 0.027 | 0.17 (0.01) | 0.083 |
| Dependent variable: total BMD |  |  |  |  |
| Age | –0.29 (0.00) | 0.008 | –0.26 (0.00) | 0.004 |
| Sex (ref: men) | –0.45 (0.03) | <0.001 | –0.39 (0.02) | <0.001 |
| DM | 0.09 (0.03) | 0.413 | – | – |
| i-PTH | –0.36 (0.00) | 0.001 | –0.32 (0.00) | 0.001 |
| 25-(OH) vitamin D | –0.03 (0.00) | 0.766 | – | – |
| Alkaline phosphatase | –0.36 (0.00) | 0.001 | –0.15 (0.00) | 0.107 |

Data are expressed as standardized β (SE). Multivariate analysis was adjusted for variables with *P* < 0.100 on univariate analysis.

Abbreviations: SE, standard error: ASM/Ht^2^, appendicular skeletal muscle mass per height squared; DM, diabetes mellitus; i-PTH, intact parathyroid hormone; 25-(OH) vitamin D, 25-hydroxy vitamin D; TMA/Ht^2^, thigh muscle area per height squared; SGA, subjective global assessment; 5STS, five times sit-to-stand test; STS30, 30-second sit-to-stand test; 6-MWT, 6-minute walk test; TUG, timed up-to-go test; BMD, bone mineral density.
