# Supplementary material for: Association Between Alkaline Phosphatase and Muscle Mass, Strength, or Physical Performance in Patients on Maintenance Hemodialysis
Source: Front Med (Lausanne). 2021 May 17;8:657957. doi: 10.3389/fmed.2021.657957 (PMC8165237; doi:10.3389/fmed.2021.657957)
Supplement: Supplementary file 3 [file Table_3.docx]

**Table S3. Correlation between ALP level and various indices according to subgroup**

|  | < 65 years (n = 61) | | ≥ 65 years (n = 23) | | Men (n = 44) | | Women (n = 40) | | Non-DM (n = 40) | | DM (n = 44) | |
| --- | --- | --- | --- | --- | --- | --- | --- | --- | --- | --- | --- | --- |
|  | *r* | *P*-value | *r* | *P*-value | *r* | *P*-value | *r* | *P*-value | *r* | *P*-value | *r* | *P*-value |
| ASM/ Ht^2^ (kg/m^2^) | –0.316 | 0.013 | –0.387 | 0.068 | –0.220 | 0.151 | –0.316 | 0.047 | –0.335 | 0.034 | –0.342 | 0.023 |
| TMA/Ht^2^ (cm^2^/m^2^) | –0.355 | 0.005 | –0.337 | 0.116 | –0.387 | 0.009 | –0.245 | 0.128 | –0.359 | 0.023 | –0.317 | 0.036 |
| BMI (kg/m^2^) | –0.177 | 0.171 | –0.433 | 0.039 | –0.360 | 0.016 | –0.199 | 0.218 | –0.265 | 0.099 | –0.232 | 0.130 |
| SGA score | –0.192 | 0.137 | –0.482 | 0.020 | –0.239 | 0.119 | –0.308 | 0.053 | –0.360 | 0.023 | –0.201 | 0.191 |
| Albumin (mg/dl) | –0.122 | 0.350 | 0.276 | 0.203 | 0.048 | 0.757 | 0.008 | 0.963 | –0.062 | 0.706 | 0.236 | 0.124 |
| Total BMD | –0.373 | 0.003 | –0.356 | 0.096 | –0.270 | 0.077 | –0.359 | 0.023 | –0.371 | 0.018 | –0.367 | 0.014 |
| HGS (kg) | –0.294 | 0.022 | –0.298 | 0.167 | –0.052 | 0.737 | –0.400 | 0.011 | –0.346 | 0.029 | –0.248 | 0.104 |
| Gait speed (m/s) | –0.395 | 0.002 | –0.182 | 0.406 | –0.268 | 0.079 | –0.322 | 0.042 | –0.341 | 0.031 | –0.338 | 0.025 |
| SPPB | –0.152 | 0.241 | –0.220 | 0.312 | 0.114 | 0.460 | –0.433 | 0.005 | –0.496 | 0.001 | –0.099 | 0.522 |
| 5STS (s) | 0.310 | 0.015 | 0.013 | 0.952 | –0.037 | 0.810 | 0.497 | 0.001 | 0.500 | 0.001 | –0.039 | 0.804 |
| STS30 (s) | –0.320 | 0.012 | –0.267 | 0.219 | –0.082 | 0.597 | –0.456 | 0.003 | –0.409 | 0.009 | –0.152 | 0.325 |
| 6-MWT (m) | –0.315 | 0.013 | –0.129 | 0.557 | –0.060 | 0.699 | –0.366 | 0.020 | –0.404 | 0.010 | –0.114 | 0.462 |
| Timed up-to-go test | 0.295 | 0.021 | 0.147 | 0.504 | 0.166 | 0.281 | 0.321 | 0.043 | 0.401 | 0.010 | 0.091 | 0.557 |

Correlation analyses were analyzed using Pearson’s correlation.

Abbreviations: *r*, correlation coefficient; DM, diabetes mellitus; ASM/Ht^2^, appendicular skeletal muscle mass per height squared; TMA/Ht^2^, thigh muscle area per height squared; BMI, body mass index; SGA, subjective global assessment; BMD, bone mineral density; HGS, handgrip strength; SPPB, short physical performance battery; 5STS, five times sit-to-stand test; STS30, 30-second sit-to-stand test; 6-MWT, 6-minute walk test.
